# The inhibitory effect against collagen-induced arthritis by *Schistosoma japonicum *infection is infection stage-dependent

**DOI:** 10.1186/1471-2172-11-28

**Published:** 2010-06-10

**Authors:** YunKun He, Jia Li, WenJia Zhuang, Lan Yin, ChunXia Chen, Jun Li, FengLi Chi, YanShuang Bai, Xiao-Ping Chen

**Affiliations:** 1 Department of Immunology, Tongji University School of Medicine, 1239 Siping Road, Shanghai 200092, China; 2 Department of Pathogen Biology, Tongji University School of Medicine, 1239 Siping Road, Shanghai 200092, China

## Abstract

**Background:**

A long-term existing schistosome infection can aid in maintaining immuno-homeostasis, thus providing protection against various types of autoimmune diseases to the infected host. Such benefits have often been associated with acute or egg stage infection and with the egg-induced Th2 response. However, since schistosome infection undergoes different stages, each associated with a specific induction of Th responses, the requirements for the ability of the different stages of schistosome infection to protect against autoimmune disease has not been elucidated. The present study was designed to study whether different stages of schistosome infection offer unique protection in collagen-induced arthritis and its mechanisms.

**Results:**

Arthritis susceptible strain DBA/1 male mice were infected with *Schistosoma japonicum *for either 2 weeks resulting in early stage infection or for 7 weeks resulting in acute or egg stage infection. Following *Schistosoma japonicum *infection, collagen II was administered to induce collagen-induced arthritis, an animal model for human rheumatoid arthritis. Infection by *Schistosoma japonicum *significantly reduced the severity and the incidence of experimental autoimmune collagen-induced arthritis. However, this beneficial effect can only be provided by a pre-established acute stage of infection but not by a pre-established early stage of the infection. The protection against collagen-induced arthritis correlated with reduced levels of anti-collagen II IgG, especially the subclass of IgG2a. Moreover, in protected mice increased levels of IL-4 were present at the time of collagen II injection together with sustained higher IL-4 levels during the course of arthritis development. In contrast, in unprotected mice minimal levels of IL-4 were present at the initial stage of collagen II challenge together with lack of IL-4 induction following *Schistosoma japonicum *infection.

**Conclusion:**

The protective effect against collagen-induced arthritis provided by *Schistosoma japonicum *infection is infection stage-dependent. Furthermore, the ability of schistosomiasis to negatively regulate the onset of collagen-induced arthritis is associated with a dominant as well as long-lasting Th2 response at the initiation and development of autoimmune joint and systemic inflammation.

## Background

The increased incidences of autoimmune diseases and atopic diseases found in developed countries [1,2] have brought the 'hygiene hypothesis' into a hot area of study and debate. The 'hygiene hypothesis' was first proposed by the British scientist Dr. Strachan in 1989 after having observed that having many siblings, especially older ones, correlated with a decreased risk of hay fever [3]. This finding has since been extended to a theory that the changed pattern in or the reduced exposure to microorganisms has led to a dysregulated immune system and hence led to increases in certain disorders like atopy and autoimmune diseases. Indeed, the mutual exclusion relationship between the incidence of immune-mediated disorders with some kinds of microbes infections, especially parasite infections, has repeatedly been reported in epidemiological studies and in animal models[4,5]. However, the requirement of the nature of parasite infection has not been fully elucidated.

Worm-like metazoan organisms so called 'helminth', including both nematoda (round worms) and platyhelminthes (flatworms), have been recognized as important infectious agents that can elicit beneficial effects to the infected host in terms of conferring resistance to atopy or autoimmune diseases. As a representative genus in parasitic platyhelminthes, schistosome or exposure to schistosome derived antigens have been found to offer protection to a range of autoimmune disorders in experimental animal models including type 1 diabetes in nonobese diabetic (NOD) mice [6,7], experimental allergic encephalomyelitis (EAE) (an animal model of multiple sclerosis) [8,9], Graves' disease [10], inflammatory bowel disease [11] and asthma [12]. However, the effect of helminth infection on collagen-induced arthritis, an animal model for human rheumatoid arthritis (RA), is less-well studied[13,14].

The immune response elicited by *Schistosoma mansoni *(*Sm*), the species that is mostly seen in Africa and South America, progresses through two phases. During the first 2-5 weeks, called early stage infection, in which the host is exposed to migrating immature parasites, the dominant response is Th1. As the parasites mature, mate and begin to produce eggs, the infection enters the acute stage during which the Th1 response decreases and the Th2 response emerges and increases. The Th2 response decreases after 12 weeks of chronic stage of the infection [15,16]. Similar immune response profiles are also found in *Schistosoma Japonicum *(*Sj*), the species mostly present in Asia [17,18].

Majority of animal studies have found that the protective effects against immune-mediated disorders provided by schistosome infection appeared to be associated with Th2 immune response induced at egg-stage or acute stage of infection. Only one study done by Osade *et al *on collagen-induced arthritis (CIA) model has demonstrated that the early stage of schistosome infection might exert any beneficial effects [14]. They found that protective effects against CIA in mice can be provided by 2 weeks *Sm *infection [14], an early stage of *Sm *infection in which eggs have not been produced in large quantities and a Th2-dominant response is usually not seen [19]. This observed protection against CIA offered at early-stage infection lacking association with a Th2 response prompted us to question whether different stages of schistosome infection would offer unique protection and whether a Th2-dominanted cytokine milieu provided by egg-stage of schistosome infection was required to achieve protective effects in CIA model. Answers to these questions will help us to better understand the mechanisms involved in parasite immune defense and use them to prevent and treat autoimmune arthritis.

In this study, we selected either a 2 or 7 weeks *Sj *infection as early or acute stages of infection to study whether different stages of *Sj *infection would affect the development of CIA differently. We found that only the 7 weeks infection regimen can offer protective or prophylactic effects against CIA, whereas the 2 weeks infection failed to provide any beneficial effect and even exacerbated the disease. Further studies indicated that the protective effects were correlated with decreased levels of anti-collagen II IgG especially the IgG2a subclass. Cytokine pattern analysis indicated that the presence of the Th2 cytokine milieu at the initiation period of CIA together with its sustained high levels was critical for the acquirement of protective effects.

## Results

### The protective effects against arthritis were only observed when a pre-established Sj infection entered acute stage; whereas an early stage of Sj infection exacerbated the arthritis

*Sm *infection can alleviate the development of several autoimmune diseases like type I diabetes [6,7] and experimental autoimmune encephalomyelitis [8,9] when the infection enters the acute stage or egg-stage of infection and when a Th2 response is dominant. To determine whether different stages of schistosomiasis would affect uniquely autoimmune arthritis in CIA model, the most widely accepted animal model for human rheumatoid arthritis (RA), and the role played by a pre-established dominant Th2 environment, DBA/1 mice were infected with the parasitic worm *Sj *either for 2 weeks for pre-established early stage of *Sj *infection (ESCIA, low Th2 response) or for 7 weeks for a pre-established acute stage of *Sj *infection (ASCIA, Th2 dominant) before the induction of CIA by collagen II (CII) immunization.

**Figure 1:**
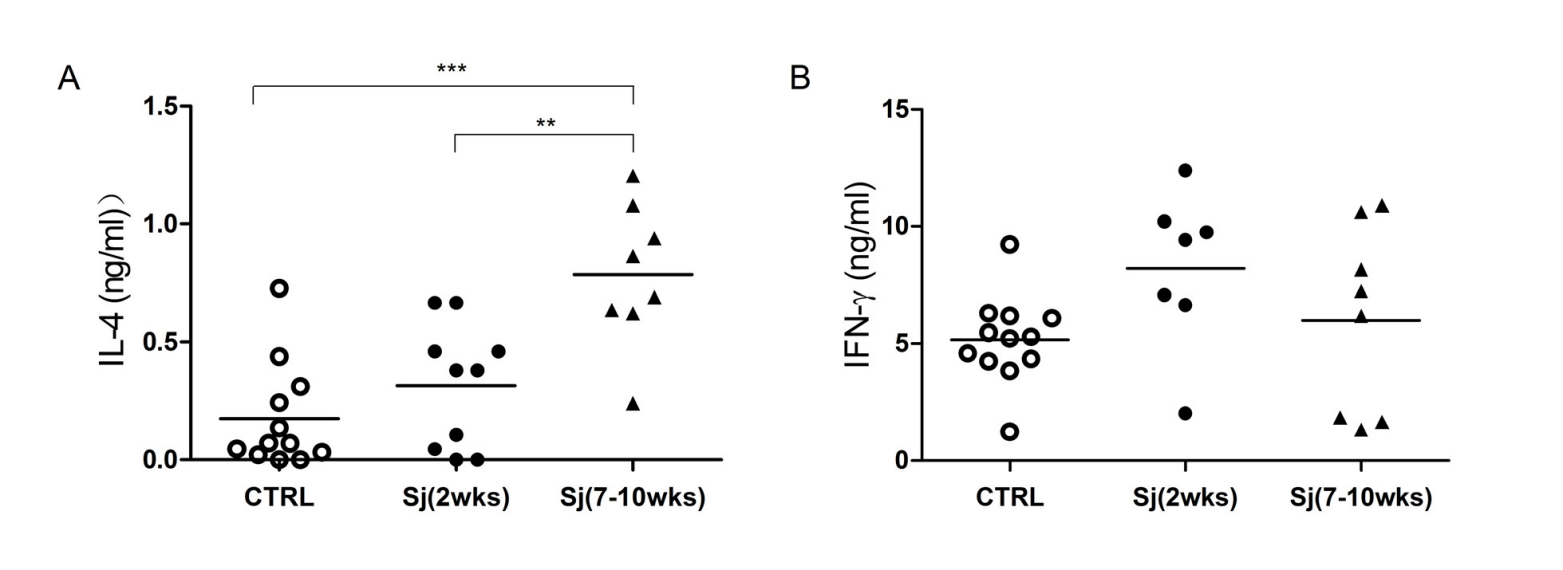
**IL-4 production is elevated in 7 to 10 weeks Sj infected DBA/1 mice**. Splenocytes from DBA/1 mice infected with Sj either for 2 weeks or 7 to 10 weeks or not infected (CTRL) were stimulated by anti-CD3 and anti-CD28 antibodies for 72 hours in vitro. The supernatants were analyzed for IL-4 (A) and IFN-g (B). Shown are the combined results from two separate experiments. Significance was tested by one-way ANOVA with Bonferroni multiple comparison test with *** and ** as p < 0.001 and p < 0.01.

As shown in Fig. 1, a significantly induced Th2 response as evaluated by a significant increase in IL-4 production by polyclonally activated spleen T cells was found in the 7 weeks infection state as compared to the 2 weeks infection state and to mice not infected. However, a slightly reduced Th1 cytokine IFN-γ production was observed in mice infected with 7 weeks compared to those infected with 2 weeks and those with no infection but without any statistical significance. Thus, consistent with the profiles found in other strains of mice infected by *Sm *or by *Sj *[16-18], DBA/1 mice also displayed Th2 dominant response when the infection enters 7 weeks but not the 2 weeks.

**Figure 2:**
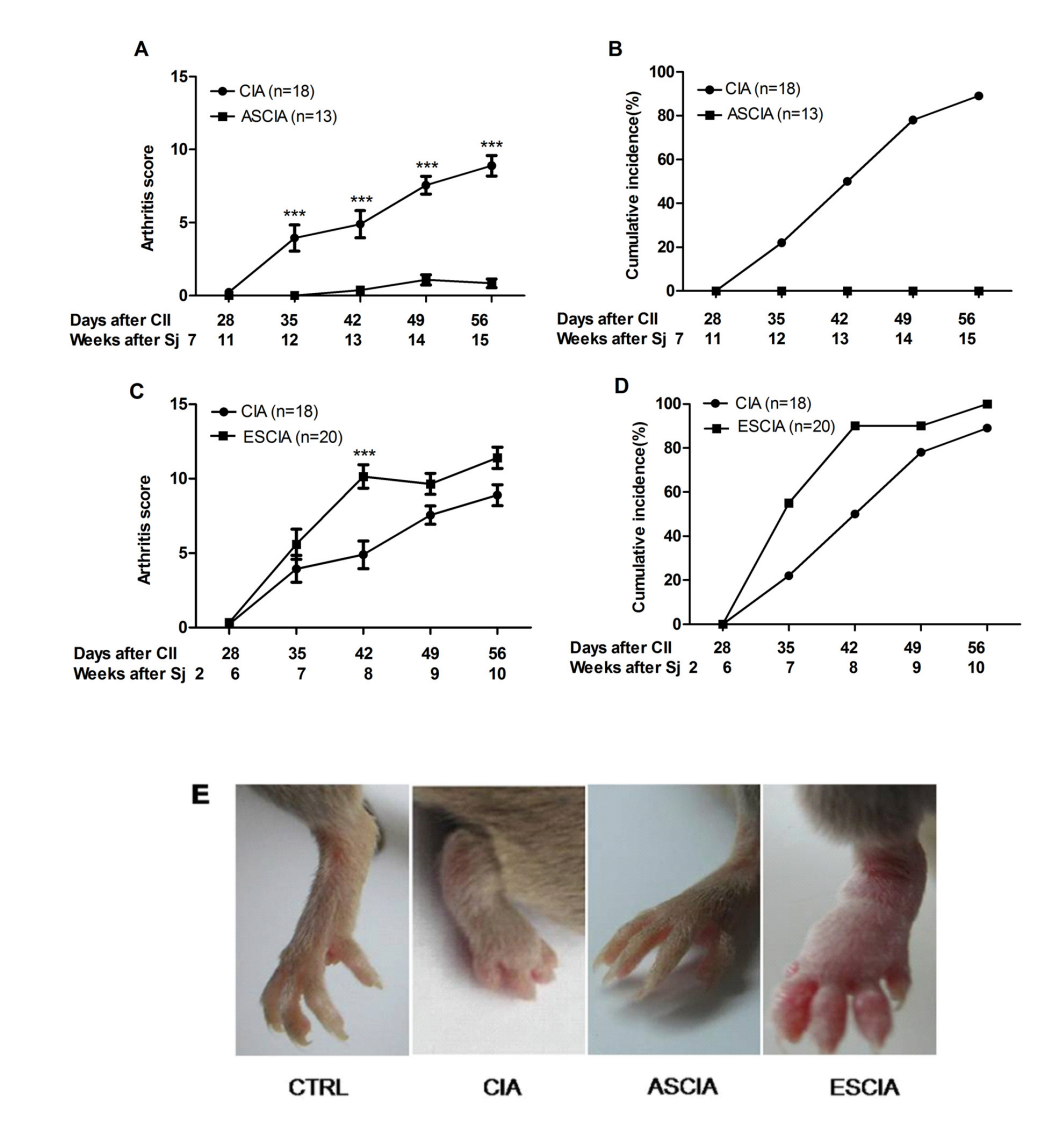
**The protective effects against arthritis were only observed when a pre-established Sj infection entered acute stage**. DBA/1 mice were infected with *Sj *for 2 weeks or 7 weeks prior to immunization with collagen II (CII) in CFA and named as ESCIA mice and ASCIA mice respectively. 7 weeks *Sj *infection significantly reduced the severity (A), incidence (B) and clinical signs (E) of arthritis in ASCIA mice; whereas 2 weeks infection by *Sj *failed to decrease the severity (C), incidence (D) and clinical signs (E) of arthritis in ESCIA mice. Arthritis was scored weekly as described in Materials and Methods. Circle and square represented uninfected CIA mice and *Sj *infected CIA mice respectively. CIA data represented the combined results from two different age groups from multiple experiments due to their similar severity and incidence. Data in ESCIA mice were the means and the standard errors of the mean (SEM) of clinical scores from 20 individual mice combined from two separate experiments, while data in ASCIA mice were from 13 individual mice combined from three separate experiments. Significance was tested by two way ANOVA with Bonferroni multiple comparison test with *** as p < 0.001. Incidence was calculated on the ratio of the number of animals with arthritis score >= 3 on one joint over total number of mice.

Arthritis was induced by application of collagen in complete Freund's adjuvant (CFA) followed by incomplete Freund's adjuvant (IFA) 21 days after first CII injection. Similar as reported [20], DBA/1 mice developed signs of arthritis around day 35 after CII immunization (Fig 2). Injection of CII in ASCIA mice which were animals infected with the 7 weeks pre-established *Sj *infection before CIA induction, disease severity was substantially reduced as assessed by ankle swelling (Fig 2E) and cumulative clinical arthritis scores of all four limbs (Fig 2A). While control CIA animals reached a cumulative arthritis score of 9 at day 56 with incidence reaching about 85%, none of the ASCIA animals developed disease (Fig 2B). In contrast, injection of CII in ESCIA mice which were animals infected with the 2 weeks pre-established *Sj *infection before CIA induction failed to provide any prophylaxis on CIA whether arthritis score (Fig 2C), arthritis incidence (Fig 2D) or clinical signs (Fig 2E) were examined. In fact, the severity of arthritis was even increased in ESCIA mice measured at day 42 (Fig 2C). As a control, non collagen-immunized mice did not develop arthritis regardless they were infected with *Sj *or not (data not shown).

### Anti-CII IgG and IgG2a levels were reduced in protected mice

**Figure 3:**
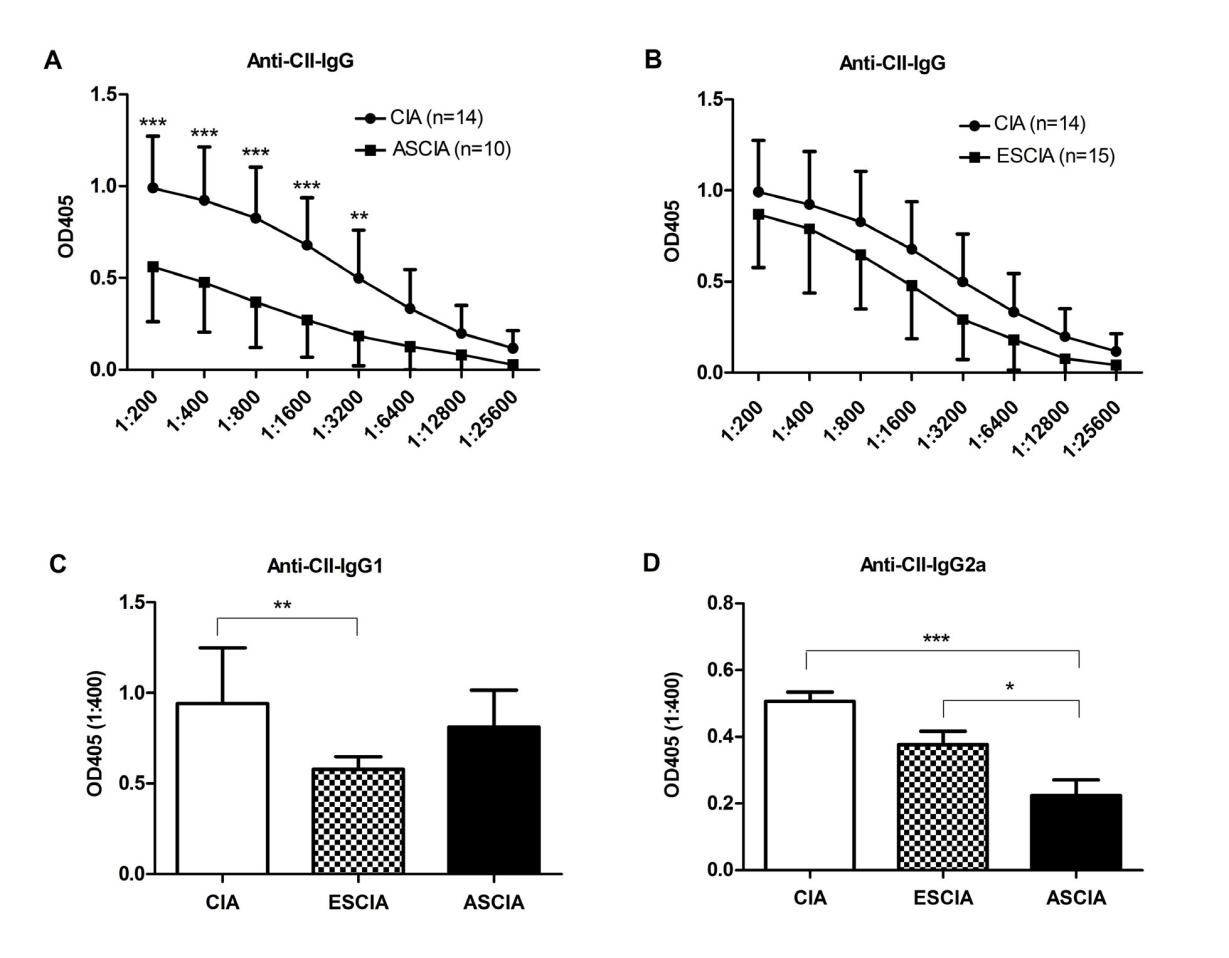
**Anti-CII IgG and IgG2a levels were reduced in protected mice**. Serums were taken from 56 days after CII challenge from ESCIA mice, ASCIA mice and CIA mice. The levels of anti-CII IgG was measured in serially diluted serum and the levels of subclasses of anti-CII IgG1 or IgG2a were measured from 1:400 diluted serum by ELISA. (A) anti-CII IgG in ASCIA vs CIA (B) anti-CII IgG in ESCIA vs CIA (C) anti-CII IgG1 (D) anti-CII IgG2a. Results were obtained from 10 to 15 serum samples collected from two or three independently performed experiments. Data presented were the mean OD ± SEM. Asterisks*** and ** represented P < 0.001 and P < 0.01 respectively by two way ANOVA for IgG or by one-way ANOVA for subclasses with Bonferroni multiple comparison test.

Anti-CII IgG has been well known for its important role in the pathogenesis of CIA and has become the marker antibody for CIA [21]. We compared the anti-CII IgG levels in protected ASCIA mice, unprotected ESCIA mice and uninfected CIA mice. As shown in Fig 3A and 3B, significantly decreased levels of anti-CII IgG were present in protected ASCIA mice as compared to uninfected CIA mice; whereas no significant difference was observed between unprotected ESCIA mice and uninfected CIA mice. This corresponded well with the prophylaxis effects observed only in ASCIA mice but not in ESCIA mice. When profiles of splenic B cells from different groups were examined, B cell percentage was found to be reduced in ASCIA mice compared to mice only challenged with CIA (36.7% vs 47.8%). However this reduction does not seem to explain the significantly decreased levels of anti-CII IgG found in ASCIA mice, because the total B cell number was not decreased due to the splenomegaly found in ASCIA mice. Furthermore, a more reduced percentage of B cells was found in ESCIA mice compared to mice only challenged with CIA (27.7% vs 47.8%), though anti-CII IgG levels remained unaffected in this group.

We further compared the levels of IgG1 or IgG2a subclasses in ASCIA mice without arthritis with those from arthritic CIA or arthritic ESCIA mice. The level of IgG2a was significantly reduced in ASCIA mice comparing to CIA or ESCIA mice (Fig 3D), corresponding to lack of arthritis. In contrast, IgG1 was reduced in ESCIA mice comparing to CIA (Fig 3C), although both groups developed arthritis comparably (Fig 2). These data suggested that IgG2a was more relevant to the pathogenesis of the arthritis, and its reduction was associated with lack of arthritis as found in ASCIA mice.

### Protective effects in ASCIA mice were associated with enhanced production of IL-4 and IL-10 and reduced production of IFN-γ

**Figure 4:**
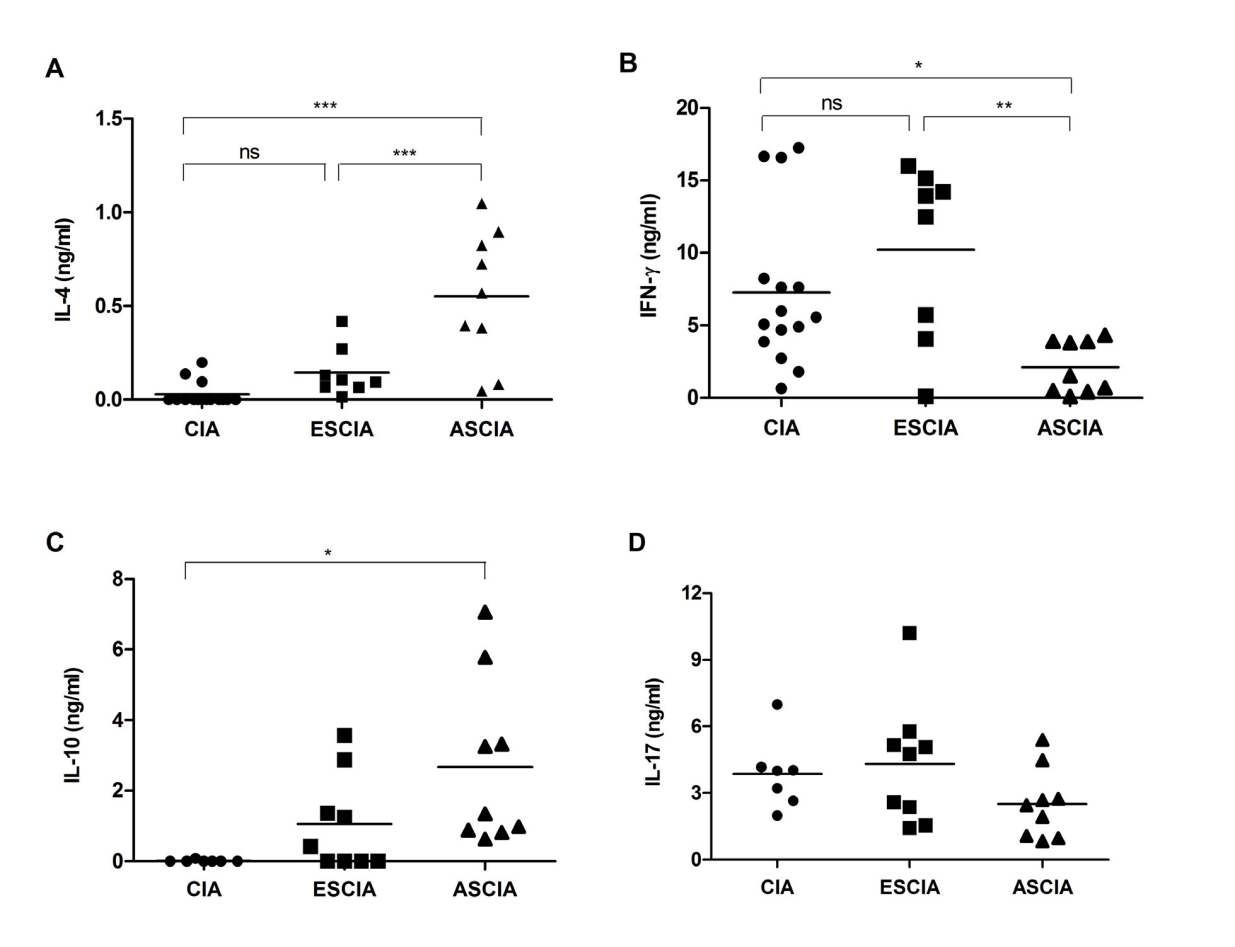
**Protective effects in ASCIA mice were associated with enhanced production of IL-4 and IL-10 and reduced production of IFN-γ**. 56 days after CII immunization in ESCIA mice, ASCIA mice and CIA mice, splenocytes were stimulated by anti-CD3 and anti-CD28 antibodies for 72 hrs. The cytokine contents for IL-4 (A), IFN-g (B), IL-10 (C) and IL-17 (D) were measured by ELISA as described in the Materials and Methods. Shown are samples from individual mouse combined from two or three separately performed experiments. Asterisks* and ** represented P < 0.05 and P < 0.01 respectively by one-way ANOVA with Bonferroni multiple comparison test.

Many studies have indicated the importance of different Th subsets and the balance among them in the pathogenesis of arthritis in the CIA model [22]. To understand whether the alleviation of arthritis in ASCIA mice was associated with altered Th responses, splenocytes were isolated at 8 weeks after CII immunization and stimulated *in vitro *with anti-CD3 and anti-CD28 antibodies. The production of IFN-γ was reduced and the production of IL-4 was enhanced in ASCIA mice compared to ESCIA and CIA mice (Fig 4A and 4B). Furthermore, moderately enhanced IL-10 levels were also found in ASCIA mice (Fig 4C). IL-17 production as induced by polyclonal stimulation of T cells demonstrated no significant differences between any of the groups (Fig 4D). Thus, an enhanced Th2 response and a reduced Th1 response appeared to correlate with the absence of disease.

**Figure 5:**
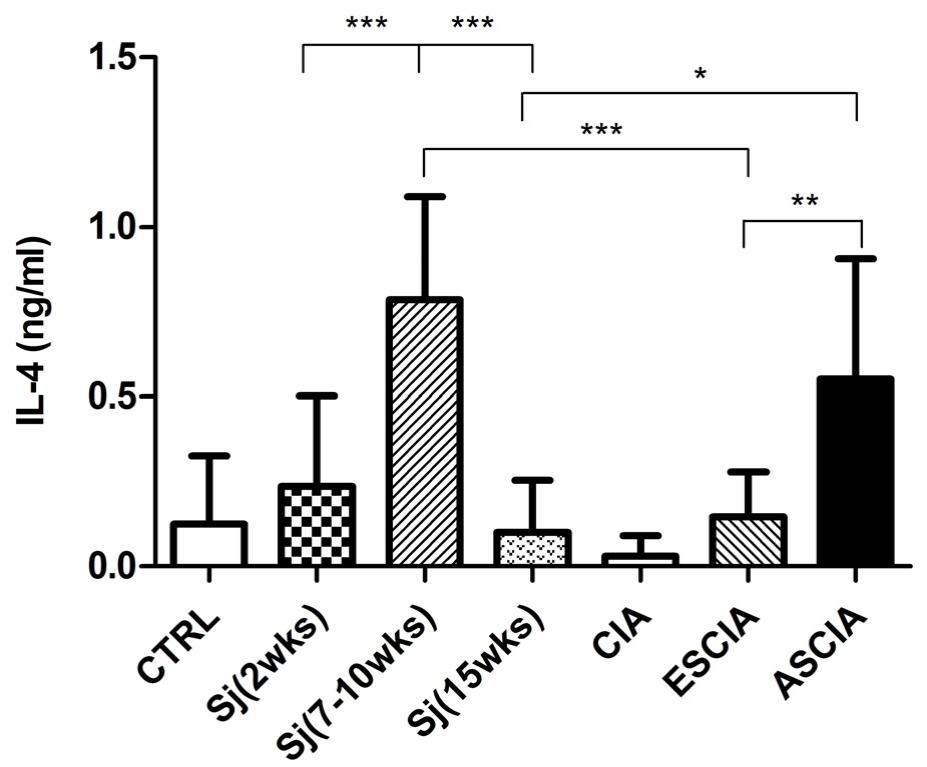
**The effects of CFA on the IL-4 production induced by Sj infection**. Splenocytes from mice of uninfected (CTRL), 2 weeks of *Sj *infection, 7 to 10 weeks of *Sj *infection, 15 weeks of *Sj *infection, CIA, ESCIA (total 10 weeks of *Sj *infection), ASCIA (total 15 weeks of *Sj *infection) were stimulated by anti-CD3 and anti-CD28 antibodies for 72 hrs. The IL-4 contents in the supernatants were measured by ELISA. Shown are samples from individual mouse combined from two or three separately performed experiments. Asterisks* and ** represented P < 0.05 and P < 0.01 respectively by one-way ANOVA with Bonferroni multiple comparison test.

The lack of significant IL-4 production in ESCIA mice (Fig 4A) led us to question whether the strong Th1-inducing agent complete Freund's adjuvant (CFA) [23] used in CIA model would alter the induction of Th2 responses induced by schistosome infection. As shown in Fig 5, the IL-4 production was low at 2 weeks of *Sj *infection, elevated at 7 to 10 weeks of infection and then decreased after 15 weeks of infection. Thus the time course of IL-4 production by *Sj *infection in DBA/1 mice is consistent with the time course found in other strains of mice infected by *Sm *or *Sj *[16,18]. However, the elevated IL-4 production occurred during 7 to 10 weeks of *Sj *infection was abrogated if CFA together with collagen was administered at 2 weeks of *Sj *infection at which IL-4 production was still low. Interestingly, CFA and collagen given at 7 weeks of infection at which IL-4 production was high prohibited the IL-4 production from being decreased when infection entered 15 week period. No differences were observed in spleen size or the appearance of the liver whether CFA was administered or not (data not shown). These results indicated that high amount of Th1-inducing agent CFA used in CIA model *per se *did not reverse an established Th2 response *in vivo *as seen in ASCIA mice which was in line with the observations made *in vitro *that an established Th2 response was irreversible [24]. Furthermore, the ability of CFA to prevent an ongoing Th2 response from being firmly established as seen in ESCIA mice which may reflect the fact that an early phase of the Th2 response is reversible [24]. More intriguingly and significantly, presence of CFA prolonged Th2 response which otherwise would be greatly reduced seen in 15 weeks infected animals. Taken together, these results indicated that a Th2 -biased milieu present at the time when CII antigen was introduced as well as during the development of arthritis correlated with lack of arthritis in protected ASCIA animals.

### FoxP3+ regulatory T cells were not different between protected and unprotected mice

It is widely accepted that FoxP3^+ ^regulatory T cells(Treg) play critical roles in pathogenesis of various of kinds of autoimmune diseases including human RA[25,26]. Both CD4^+^CD25^+ ^T cells and CD4^+^CD25^+^FoxP3^+ ^T cells in the spleen were measured and compared. The correspondence of these two populations of T cells was about 80-90% (Table 1). There was a slightly increased representation of FoxP3^+ ^Treg during infection, whether it was CIA mice alone or *Sj *infection with CIA mice. However there was no remarkable increase in FoxP3^+ ^Treg in ASCIA mice without arthritis compared to CIA or ESCIA mice with arthritis.

**Table 1:** CD4+CD25+FoxP3+ regulatory T Cell compartment in the spleen

Mouse Group	%CD4(SD)	
	CD4+CD25+	CD4+CD25+FoxP3+
Normal	8.9(0.3)	7.4(0.9)
CIA	10.9(2.0)	9.3(3.1)
ESCIA	10.9(1.6)	7.7(0.8)
ASCIA	12.9(2.2)	10.5(1.9)

The number of mice per group is from 8 to 13 from two or three separately performed experiments. Each value was derived from the means of individual mouse in the group.

## Discussion

Helminth parasites inhabit immune-competent hosts for long periods of time and appear to develop strategies to induce strong anti-inflammatory responses in the infected host. Loss of natural helminth exposure as a result of improved hygiene and wide-use of anti-helminth drugs result increased incidences of immune-mediated disorders evidenced by epidemiological studies. On the other hand, the helminths provide the host with more balanced immuno-homeostasis and demonstrate beneficial effects against various kinds of autoimmune diseases in animal studies[4]. In fact several clinical trials have attempted to use helminth to treat autoimmune disease in patients and showed that exposure to helminths can reduce disease activity in patients with immune-mediated disease like ulcerative colitis[5]. In this study, we investigated the beneficial effect of schistosome infection on less-well studied collagen-induced arthritis mouse model. The beneficial effect is found to be associated with reduced anti-collagen II IgG production and with a Th2 cytokine environment induced at egg-stage of infection.

The anti-CII antibodies have been recognized as important pathogenic factors in the initiation and development of CIA in mice. It has been reported that arthritis can be transferred passively to naive animals with CII-reactive serum [27], and by monoclonal antibody to CII [28]. In fact, a different mouse model collagen antibody-induced arthritis (CAIA) for human RA is established by passively transferring arthritogenic antibodies against collagen. Further studies using the CAIA model found that the subclass of IgG2a appears to be more efficient in inducing RA probably due to its strong capacity to activate complement through the classical pathway [29]. The ability of inhibiting arthritis by egg-stage *Sj *infection in ASCIA mice but not by early stage infection found in our study was well correlated with reduced levels of anti-CII IgG, especially the levels of IgG2a. This alteration in class switch may reflect the significantly repressed IgG2a-promoting cytokine IFN-γ production found in ASCIA group. On the other hand, the positive association between elevated IgG2a and the occurrence of arthritis indicates the pathogenic role played by Th1 cells and the beneficial role played by Th2 cells. Consequently, beneficial effects against CIA can only be found in the acute or egg-stage of *Sj *infection but not in early stage infection before eggs are produced in large quantity.

Schistosomiasis is a well-characterized Th2 response-dominated disease [16]. Shortly after the beginning of egg deposition, a strong egg-specific Th2 response develops characterized by high levels of IL-4, IL-5, and IL-13 which decreases when the infection enters the chronic stage [17,30]. Not surprisingly the negative effect of schistosome infection on the induction and development of type I diabetes in NOD model [6], multiple sclerosis in EAE model [8] and in mouse model of Graves' hyperthyroidism [10] has repeatedly been reported in mice infected by schistosme for 6-8 weeks before autoimmune diseases were induced. By comparison between the beneficial effects of *Sj *infection and the IL-4 production profiles, our study clearly demonstrated that the presence of Th2 at the beginning stage of autoimmune attack was important in conferring the protection. In addition, our study also demonstrated a positive correlation between the prolonged presence of elevated Th2 levels during the development stage of autoinflammatory response and the absence of disease.

We noticed the different result obtained by Osada *et al *[14] in which 2 weeks prior Schistosome infection was shown to offer protection in CIA. An obvious difference between our study and that of Osada's was that different strains of Schistosoma were used, ie, *Sm *in their study and *Sj *in ours. Since the amount of eggs along with the levels of Th2 response induced are low after 2 weeks *Sm *infection [19], Osada's study seems to suggest that the Th2 milieu present at initiation phase of autoimmune attack is not crucial to achieve the protection. In addition, elevated IL-4 production was still found to be produced by activated splenocytes from infected and CFA injected mice, suggesting that the injection of the Th1-promoting agent CFA in their study has little impact on the development of the Th2 response. It is not clear at present that if different strains of worms used can fully explain the different results obtained.

Another possible protective mechanism involved in our study was likely associated with the enhanced production of regulatory cytokine IL-10 by activated T cells found in protected animals. Th2 cells, FoxP3^+ ^CD4^+ ^Treg cells and FoxP3^- ^IL-10-producing CD4^+ ^so called Tr1 cells[31] may all contribute to this induced production of T cell-derived IL-10. Although no significant increase in Treg numbers was found in protected group, the possibility that the activities of Treg and Tr1 cells were enhanced can not be excluded. No significant difference on production of IL-17 was found among protected mice, unprotected mice and CIA mice, even though Th17 appears to be accepted as the more critical pathogenic Th cells in the CIA model than Th1 [32].

Our study also provided an approach to explore the plasticity of Th subsets *in vivo. In vitro *studies have demonstrated that Th17 is an unstable subset [33], whereas Th1 and Th2 subsets, once they are well established, are not reversible [24]. With the application of Th1-promoting agent CFA at different time point, we found that CFA together with collagen is able to inhibit the Th2 response only at 2 weeks but not at 7 weeks of *Sj *infection when Th2 development is at an early stage but not well-established. Thus our results for the first time *in vivo *indicated that a well established Th2 response can not be reversed, whereas an early stage Th2 response can be reversed in the presence of CFA treatment. This phenomenon may serve as the basis for the beneficial effects by *Sj *infection in maintaining immune-homeostasis in infected host hence providing protection against immune-mediated diseases.

## Conclusion

In summary, we demonstrated in this study that *Schistosoma japonicum *infection like *Schistosoma masoni *can inhibit the incidence and severity of autoimmune arthritis in the mouse CIA model. However this effect is absolutely infection stage-dependent, *ie*, it occurs at acute stage of infection but not at early stage of infection. Furthermore this beneficial effect is positively associated with the presence of an irreversibly established Th2 response at the initiation of autoimmune response and with its sustained high levels during the development of arthritis. Thus our study provides more insight into the complex mechanisms regarding to the "hygiene hypothesis" and the potential difficulties faced when egg or egg-derived materials are used for therapeutic agents in Th1-associated autoimmune diseases.

## Methods

### Mice

6-12 weeks old male DBA/1 mice were purchased from Songjiang Animal Facility of the Chinese Academy of Sciences of Shanghai. All mice were maintained under specific pathogen-free conditions and fed with standard laboratory food and water. All procedures performed on animals within this study were conducted in accordance with and by approval of the Internal Review Board of Tongji University School of Medicine.

### *Schistosoma japonicum *Infection in mice

DBA/1 mice were infected by percutaneous exposure of the abdomen with 20 cercariae per mouse. Cercariae of *Sj *used for all experiments were obtained from Oncomelania hupensis snails collected from Guichi County, Anhui Province, and maintained at the National Institute of Parasitic Diseases, Shanghai. Uninfected animals of the same sex and age were maintained as controls. At the end of each experiment, the livers and spleens from infected mice were routinely examined to ensure that schistosomiasis was established.

### Induction and assessment of CIA

CIA was induced according to the methods as described by Current Protocol in Immunology [34]. Chicken type II collagen(CII) (Sigma) was dissolved in 0.05 M acetic acid at concentration of 2 mg/ml by stirring overnight at 4°C and was then emulsified in an equal volume of complete Freund's adjuvant (CFA) containing 4 mg/ml Mycobacterium tuberculosis (Chondrex, Redmond, WA). Male DBA/1 mice were injected intradermally at the base of the tail with 0.1 ml of emulsion containing 100 μg of collagen II. 21 days after primary immunization, mice were boosted with 0.1 ml of the mixture of 1 mg/ml CII emulsified in incomplete Freund's adjuvant (Sigma) via the same route. In order to keep the age of animals comparable for arthritis profiles, different ages of mice were selected for experiments for either early or acute infection. For experiments of early stage of infection, mice of 10-12 weeks old were used with first injection of CII at 2 weeks after *Sj *infection, named ESCIA mice. For experiment of acute stage of infection, mice of 6-8 weeks old were used with first injection of CII at 7 weeks after *Sj *infection, named ASCIA mice.

Arthritis was scored as described in Current Protocol in Immunology [34] from day 21 using a scale of 0-4 per limb in which 0 = No evidence of redness and swelling; 1 = Redness and mild swelling confined to the mid-foot or ankle joint; 2 = Redness and mild swelling extending from the ankle to the mid-foot; 3 = Redness and moderate swelling extending from the ankle to the metatarsal joints; 4 = Redness and severe swelling encompass the ankle, foot and digits. The total arthritis score in one mouse is the sum of scores of all four feet with maximum score of 16. The incidence of CIA was calculated based on the number of mice with at least one foot scored higher than 3.

### Measurement of antibodies against CII

The levels of anti-CII IgG or its subclasses in serum were measured by ELISA. Serum was collected at day 56 after first CII immunization. In brief, 96-well ELISA microplates (Greiner) were coated with CII at 5 μg/ml dissolved in 0.1 N NaHCO3, pH9.6 buffer at 100 μl/well at 4°C overnight. 100 μl of diluted serum sample was incubated at 4°C overnight. The plates were washed with PBST (0.5% Tween-20 in PBS) three times, followed by adding peroxidase conjugated goat anti-mouse IgG at 1:2000 (CapitalBo Corp., Beijing) or peroxidase-conjugated anti-mouse IgG1 or anti-mouse IgG2a at 1:10000 (Santa Cruz) at 100 μl/well. After 2 hr incubation at room temperature and wash, the final color development was achieved by adding peroxidase substrate ABTS (2,2'-Azino-bis(3-Ethylbenzthiazoline-6-Sulfonic Acid) , Sigma) to each well at 100 μl/well and the absorbance was measured at 405 nm at appropriate time.

### Measurement of cytokine production by splenocytes

To study cytokine expression by T lymphocytes, isolated spleen cells (5 × 10^6 ^cells/well) from mice that were immunized by CII for 8 weeks or from control mice were stimulated with 1 μg/ml anti-CD3ε (eBioscience) and 1 μg/ml anti-CD28 (eBioscience) at 37°C, 5%CO2 in 10% FCS/RPMI(GIBCO) culture medium for 72 hrs. The cytokine contents in the supernatants were analyzed by ELISA analysis as described by the e-Bioscience protocol http://www.ebioscience.com. All coating and detection antibodies were purchased from e-Bioscience (Dakewe, Shanghai), and recombinant mouse cytokines were obtained from Peprotech (Dakewe, Shanghai) for standard curves in ELISA assay.

### Flow cytometric analysis of regulatory T cell, T cell and B cell

The levels of regulatory T cell (Treg) in splenocytes were measured by flowcytometry analysis as instructed by the Mouse Regulatory T cell Staining Kit (eBioscience). T cell and B cell were detected by FACS analysis staining with either FITC-anti-CD3ε (eBioscience) or PE-anti-B220 (eBioscience). All data were collected on a FACSCalibur (Becton Dickinson) and analyzed with FlowJo software (Tree Star, Ashland, OR).

### Statistical analyses

Differences in arthritis score and differences in antibody levels of anti-CII IgG were analyzed with two-way ANOVA with Bonferroni multiple comparison tests. Differences in levels of cytokine production and subclasses of IgG were analyzed by one-way ANOVA with Bonferroni multiple comparison test. Calculations for statistical analysis were performed with Prism software. P < 0.05(*), P < 0.01(**) and P < 0.001(***) were considered as statistically significant.

## Abbreviations

Sj: Schistosoma japonicum; Sm: Schistosoma masoni; CII: type II collagen; CIA: collagen-induced arthritis; CFA: complete Freund's adjuvant; ELISA: enzyme linked immunosorbant assay; IFN: interferon; IL: interleukin.

## Authors' contributions

YKH, JL, YL and XPC participated in the design of the study. JL did the initial work and YKH performed majority of the experiments. CXC, FLC and YSB recorded the arthritis. JL and WJZ did the infection. YKH and XPC participated in the statistical analysis. XPC wrote the manuscript. All authors read and approved the final manuscript.
